# Glutamate Prevents Altered Mitochondrial Function Following Recurrent Low Glucose in Hypothalamic but Not Cortical Primary Rat Astrocytes

**DOI:** 10.3390/cells11213422

**Published:** 2022-10-29

**Authors:** Paul G. Weightman Potter, Kate L. J. Ellacott, Andrew D. Randall, Craig Beall

**Affiliations:** Department of Clinical and Biomedical Sciences, Faculty of Medicine and Health, University of Exeter Medical School, RILD Building, Barrack Road, Exeter EX2 5DW, UK

**Keywords:** astrocytes, hypoglycemia, diabetes mellitus, type 1, mitochondria, glycemic control, hypothalamus, glutamic acid

## Abstract

Astrocytes contribute to glutamatergic signalling, which is required for hypoglycaemia counterregulation and is impaired by recurrent insulin-induced hypoglycaemia. This study examined the glutamate response of astrocytes when challenged with acute and recurrent low glucose (RLG) exposure. The metabolic responses of cortical (CRTAS) and hypothalamic (HTAS) primary rat astrocytes were measured in acute and recurrent low glucose using extracellular flux analyses. RLG caused mitochondrial adaptations in both HTAS and CRTAS, many of which were attenuated by glutamate exposure during low glucose (LG) treatments. We observed an increase in capacity of HTAS to metabolise glutamine after RLG exposure. Demonstrating astrocytic heterogeneity in the response to LG, CRTAS increased cellular acidification, a marker of glycolysis in LG, whereas this decreased in HTAS. The directional change in intracellular Ca^2+^ levels of each cell type, correlated with the change in extracellular acidification rate (ECAR) during LG. Further examination of glutamate-induced Ca^2+^ responses in low glucose treated CRTAS and HTAS identified sub-populations of glucose-excited- and glucose-inhibited-like cells with differing responses to glutamate. Lastly, release of the gliotransmitter ATP by HTAS was elevated by RLG, both with and without concurrent glutamate exposure. Therefore, hypothalamic astrocytes adapt to RLG by increasing glutamate uptake and oxidation in a manner that prevents RLG-induced mitochondrial adaptations.

## 1. Introduction

Recurrent hypoglycaemia (RH) remains a major barrier to optimal glucose control for people with diabetes [1]. Acutely, hypoglycaemia blunts the counterregulatory responses (CRR) to future hypoglycaemia culminating in impaired hypoglycaemia awareness [2]. The aetiology of impaired hypoglycaemia awareness, however, is not fully understood. The counterregulatory response to hypoglycaemia is, in part, orchestrated by the central nervous system, which either directly senses, or receives inputs regarding peripheral glucose levels and consequently mediates behavioural and hormonal responses such as suppression of insulin and increases in glucagon and adrenaline release. The circumventricular organs also facilitate sensing of circulating factors such as hormones and other nutrients [3]. Specifically, the hypothalamus and hindbrain contain nuclei important for glucose-sensing, containing both glucose-excited (GE) and glucose-inhibited (GI) neurons. In addition to neurons, astrocytes are also critical for detection of mild hypoglycaemia. In a mouse model with a whole-body knockout of glucose transporter 2 (GLUT2), the selective re-expression of GLUT2 in astrocytes was sufficient to restore CRR to hypoglycaemia [4], this outcome was not repeated when GLUT2 was reintroduced to neurons. However, GLUT2-expressing neurons in the NTS also contribute to hypoglycaemia-detection [5]. Additionally, in ex vivo brain slices containing the nucleus tractus solitarius (NTS), raised intracellular Ca^2+^ concentrations in response to low glucose were observed in astrocytes before neurons [6,7,8]. Furthermore, selective blockade of astrocyte metabolism using fluorocitrate prevents neuronal activation in response to low glucose [7,9]. Together these data highlight that astrocytes are integral for glucose-sensing and facilitate the CRR to hypoglycaemia in partnership with neurons. How recurrent hypoglycaemia affects astrocytic function in CRR remains unclear.

According to the alternative fuel hypothesis, the brain adapts to utilise substrates other than glucose to maintain neuronal function and thus becomes less sensitive to changes in glucose. For example, neuronal activity in ex vivo hippocampal brain slices can be maintained in the absence of glucose by the supply of lactate [10]. In people with type 1 diabetes, monocarboxylic acid (acetate) transport can maintain brain activity during hypoglycaemia [11]. In people with hypoglycaemia unawareness, increased brain lactate levels have been reported [12]. Human astrocytes in vitro metabolically adapt to recurrent low glucose (RLG), to become more dependent on fatty acid oxidation (FAO), providing further evidence of metabolic fuel switching [13].

During hypoglycaemia, glutamatergic signalling increases in the ventromedial hypothalamus (VMH) and is required for CRR [14]. Mice lacking a functional vesicular glutamate transporter 2 (*Vglut2*) gene in VMH steroidogenic factor 1 (SF1) neurons have impaired hypoglycaemia counterregulation [15]. In the VMH of rats exposed to hypoglycaemia, interstitial glutamate levels increase [14], but following recurrent hypoglycaemia, the levels fail to rise indicating impaired glutamatergic signalling. A well-understood role of astrocytes at a glutamatergic synapse is to sequester and recycle glutamate from the synaptic cleft and release this back to neurons as glutamine. Approximately 80% of glutamate released in a synapse is sequestered by astrocytes via glutamate transporter 1 (GLT-1; EAAT2) and glutamate/aspartate transporter (GLAST; EAAT1) [16,17]. This requires the co-transport of three Na^+^ ions per glutamate molecule. To maintain ionic homeostasis, Na^+^ ions are extruded from the cell by the both Na^+^/Ca^2+^ exchanger and the Na^+^/K^+^-ATPase. Approximately 20% of astrocytic ATP production is utilised for maintaining Na^+^/K^+^-ATPase activity, making glutamate recycling an energy-intensive process [18]. Additionally, astrocytes also express ionotropic [19] and metabotropic [20] glutamate receptors which allow movement of ions such as K^+^, Na^+^, and Ca^2+^ or the release of Ca^2+^ from the endoplasmic reticulum. Maintenance of Ca^2+^ homeostasis is also ATP-dependent requiring the activity of the plasma membrane Ca^2+^-ATPase (PMCA) and sarco/endoplasmic reticulum Ca^2+^-ATPase (SERCA) to pump Ca^2+^ out of the cell or return them into the endoplasmic reticulum, respectively, [21].

The substantial energy requirements of glutamate recycling, place a significant demand upon astrocyte mitochondrial and glycolytic energy production [22]. This presents a combined challenge in the context of hypoglycaemia where astrocytes, with limited glucose availability, still need to sustain glutamate-glutamine recycling to support neuronal function. In addition to glucose, astrocytes can also utilise other substrates to meet their energetic demands. Astrocytes contain glycogen which is broken down during times of low glucose (LG) availability to maintain ATP production and lactate to export to neurons to provide a metabolic substrate [23,24]. Additionally, astrocytes metabolise glutamate as fuel via the tricarboxylic acid (TCA) cycle [25]. Interestingly, as both glutamate levels increase or glucose availability decreases more glutamate is shuttled into the TCA cycle [26,27].

The studies detailed here aimed to characterise for the first time how rat astrocytes from the hypothalamus and cortex respond to glutamate challenge during or following exposure to acute and recurrent low glucose levels. Within cell populations derived from each brain region, different astrocyte subtypes were identified on a single cell scale and their responses to acute LG and glutamate were characterised. On a population level, the mitochondrial respiration and glycolytic activity of astrocytes from the cortex and hypothalamus were measured during acute LG and RLG with and without glutamate. These studies test the hypothesis that rat primary astrocytes display altered metabolism after RLG exposure and adaptations are exacerbated by glutamate co-exposure during energy stress.

## 2. Materials and Methods

### 2.1. Animals

All animal studies were conducted in accordance with the UK Animals in Scientific Procedures Act 1986 (ASPA) and study plans were approved by the institutional Animal Welfare and Ethical Review Body at the University of Exeter. Cells derived from neonatal (p1-5) Sprague Dawley rats (Charles River, Margate, UK) were used for all experiments. Rats were group housed on a 12:12 light–dark cycle at 22 ± 2 °C, with unlimited access to standard laboratory rodent diet (EURodent diet [5LF2], LabDiet, London, UK) and water.

### 2.2. Astrocyte Isolation and Cell Culture

Following decapitation, primary rat astrocytes were isolated (see extra supplementary materials; ESM; methods for details) from the cortex and hypothalamus of neonatal rats P1-5. Cultures were generated by pooling multiple cortices/hypothalami and validated as astrocyte-enriched cultures of >90% GFAP-immunoreactive cells by manual counting and confirmed as expressing both excitatory amino acid transporters EAAT1 and EAAT2 (ESM Figure S1). Cortical-enriched astrocytes (CRTAS) and hypothalamic-enriched astrocytes (HTAS) were cultured in stock media (DMEM; ThermoFisher Scientific, Oxford, UK) containing 10% foetal bovine serum (*v*/*v*) (Seralabs, Burgess Hill, UK), L-glutamine (4 mmol/L); penicillin/streptomycin (100 U/mL; 100 µg/mL, ThermoFisher Scientific), and 7.5 mmol/L D-glucose. Cells were cultured for 5–10 days before being plated for experiments. For 24–72 h before experiments took place, cells were cultured with dibutyryl-cyclic-adenosine monophosphate (d-cAMP; Santa Cruz, Dallas, TX, USA; 200 µmol/L) to improve astrocyte maturity [28]. On the experimental day, cells were cultured in 2.5 mmol/L glucose-containing medium for 2 h to progressively lower glucose concentrations (for seeding densities used see ESM methods). For acute treatments, the medium was replaced with either 2.5 or 0.1 mmol/L glucose containing media and maintained for 30–180 min. The RLG protocol was performed as previously described [13], with minor modifications. Cells were recovered in stock media, containing 7.5 mmol/L glucose and d-cAMP (200 µmol/L) overnight. This included three days of control or RLG with and without glutamate (100 µmol/L), followed by the fourth day which included either 2.5 or 0.1 mmol/L glucose with and without glutamate creating four groups; control (C), control with glutamate (C + glut), RLG, and RLG with glutamate (RLG + glut) (ESM Figure S2).

### 2.3. Ratiometric Calcium Imaging

Single-cell Ca^2+^ imaging was performed using the ratiometric cell-permeant dye Fura-2 AM (Life Technologies, Renfrew, UK). (ESM, supplementary methods). Briefly, cells were incubated in serum-free DMEM containing 2.5 mmol/L glucose for 1 h at 37 °C before being loaded with Fura-2 AM (4 μmol/L; Life Technologies) for one hour in HEPES-buffered balanced salt solution (in mmol/l: 135 NaCl, 5 KCl, 2 CaCl_2_, 1 MgCl_2_, 10 HEPES, pH 7.4) supplemented with either 2.5 or 0.1 mmol/L glucose depending on the experimental paradigm. Before imaging began, Fura-2 AM-containing normal saline was removed and replaced with fresh saline containing the same glucose concentration. Imaging was performed using a TE 2000-S Eclipse microscope (Nikon, Surbiton, UK), using the 79001-ET Fura2 filter set (Chroma Technology Corporation, Bellows Falls, VT, USA), the Lambda DG4 light source (Sutter Instrument Company, Novato, CA, USA), and an ORCA-ER digital camera (Hamamatsu Photonics, Welwyn Garden City, UK). Throughout imaging, cells were continuously perfused in a low volume chamber and ratiometric Ca^2+^ imaging was performed using pair-wise exposures at 340 and 380 nm and analysed using Volocity software (v5.5; Perkin Elmer; Beaconsfield, UK). For 1 h-long low glucose exposure recordings, the cells were maintained in 2.5 mmol/L glucose for 5 min. The glucose concentration was dropped to 0.1 mmol/L or maintained in 2.5 mmol/L for 1 h. In the glucose reperfusion studies, the normal glucose-treated cells were loaded with Fura-2 AM and imaged at 2.5 mmol/L. Separate dishes were loaded with Fura-2 AM in 0.1 mM glucose and Ca^2+^ signals measured for 6 min before the glucose concentration was raised to 2.5 mmol/L. After 65 min of imaging glutamate (100 µmol/L) was perfused for 3 min in saline containing the same glucose concentration as the preceding 1 h. After which the cells were washed with HEPES-buffered balanced salt solution for 5 min.

For analysis the experimental unit was an individual cell and only glutamate-sensitive cells were counted: defined as having a greater than or equal to 10% increase in intracellular Ca^2+^ concentration during the 3 min glutamate exposure. Every five minutes, one minute of data was binned. A rate of change of larger than the mean delta of normal glucose-treated cells ± 2.5 standard deviations was designated as glucose-sensing. Cells with an increase in intracellular Ca^2+^ concentration ([Ca^2+^]_i_) during low glucose exposure were classified as glucose-inhibited-like (GI-like) cells, conversely if [Ca^2+^]_i_ decreased in low glucose, they were designated as glucose-excited-like (GE-like). For glucose reperfusion experiments where glucose was increased from low to normal, the [Ca^2+^]_i_ of a GE-like cell increases whereas [Ca^2+^]_i_ in a GI-like cell decreases.

### 2.4. Analysis of Cellular Metabolism

The Seahorse XF^e^96 analyser was used to determine the oxygen consumption rate (OCR) and extracellular acidification rate (ECAR) of rat primary astrocytes as previously described, with minor modifications [13]. Cells were seeded at 2 × 10^4^ per well of a Seahorse XF^e^96 assay plate (102416-100, Agilent, Cheadle, UK), the day before the study. The medium was exchanged with low buffered media, containing 2.5 or 0.1 mmol/L glucose, and incubated for 1 h in atmospheric CO_2_ at 37 °C. Mitochondrial stress tests (no. 103015-100, Agilent) and mitochondrial fuel flexibility tests (no. 103270-100, Agilent) were performed as per manufacturer’s instructions (see ESM Methods for further details). For analysis purposes, wells with negative values for OCR were excluded from the analysis and the experimental unit was each independent test or well.

### 2.5. Measurement of Intracellular and Extracellular Glutamate and Glutamine

The Glutamine/Glutamate-Glo™ assay (J8021; Promega, Southampton, UK) was used as per the manufacturer’s instructions and as described previously [29]. Briefly, CRTAS and HTAS were exposed to RLG with and without glutamate and on the third day, seeded into a 96 well plate at 2 × 10^4^ per well. On the fourth day, cells were incubated in glutamine-free DMEM (#A1443001, ThermoFisher Scientific) with normal or low glucose, ±glutamate, for 1 h before conditioned media and cell lysates were collected. For data analysis the experimental units were cells generated from separate cultures of astrocytes.

### 2.6. Measurement of Glutamate Dehydrogenase Enzymatic Activity

Glutamate dehydrogenase (GDH) activity was measured in cell samples of CRTAS and HTAS exposed to normal glucose levels in the absence of glutamate (control), control plus glutamate (C + glut), recurrent low glucose (RLG), or RLG plus glutamate (RLG + glut) conditions for 30 min. The assay (#ab102527, Abcam, Cambridge, UK) was performed following the manufacturer’s instructions. GDH activity was measured as a colourimetric change (λmax = 450 nm) using a PHERAstar Fs (BMG Labtech, Ortenberg, Germany). For data analysis the experimental units were cells generated from separate cultures of astrocytes.

### 2.7. Quantifying Intracellular and Extracellular ATP Levels

Total and extracellular ATP levels were measured using ATPlite (no. 6016941, Perkin Elmer, Seer Green, UK) with minor modifications, as previously described [30]. Briefly, total ATP levels were calculated from cells seeded at 1 × 10^3^ per well of a white-walled 96 well plate and exposed to phenol red free DMEM containing 2.5 or 0.1 mmol/L glucose with glutamate (100 µmol/L) for 30 min. Extracellular ATP levels were measured from conditioned medium collected from 60 mm dishes containing cells exposed to 2.5 or 0.1 mmol/L glucose with and without glutamate (100 µmol/L) for 30 min. For data analysis the experimental units were cells generated from separate cultures of astrocytes.

### 2.8. Measurement of Extracellular Lactate

Lactate was measured in conditioned media samples of CRTAS and HTAS exposed to control, C + glut, RLG, or RLG + glut conditions for 30 min in 60 mm dishes. The assay (#ab65331, Abcam, UK) was performed following the manufacturer’s instructions. Briefly, in the presence of lactate dehydrogenase, lactate was oxidised to generate a product which interacts with a probe to produce a colour (λmax = 450 nm) which was quantified colourimetrically by PHERAstar Fs. Values were normalised to the control sample within the set. For data analysis the experimental units were cells generated from separate cultures of astrocytes.

### 2.9. Fluorescent Imaging

Mitochondrial morphology was examined by staining CRTAS and HTAS exposed to control, C + glut, RLG, or RLG + glut cells in the presence of glutamate (100 µmol/L). Cells were stained with MitoTracker Red CMXRos (50 nmol/L; M7512, no. 1785958, ThermoFisher Scientific, Oxford, UK) before fixing and imaging using confocal microscopy (Leica, London, UK DMi8; ×63/oil immersion lens) by an investigator blind to sample identity. See ESM Methods for further details. To confirm enrichment of astrocytes in primary cultures, once confluent, astrocytes were seeded onto coverslips and fixed by the addition of −20 °C methanol for 24 h. Cells were stained for GFAP and DAPI. The number of GFAP positive cells was greater than or equal to 90% in the cultures tested (ESM Figure S1).

### 2.10. Statistical Analyses

For comparisons of two groups of normally distributed data, unpaired t-tests were used. For data that were not normally distributed, Mann–Whitney U tests were used. For comparisons of multiple groups, normally distributed data were analysed with a one-way analysis of variance (ANOVA) with post hoc Tukey’s multiple comparisons test, and abnormally distributed data were analysed using a Kruskal–Wallis test with post hoc Dunn’s tests. Outliers were also detected and excluded using the ROUT method. Area under the curve (AUC) analysis was also performed using the trapezoid rule on the total area of the curves. To measure decay, the time constant (tau, τ) was used which represents the time it would take for signal to reach zero given sufficient time. Statistical analyses were performed using GraphPad Prism software (Prism v9.2.0 (332); GraphPad Software, La Jolla, CA, USA). Results are expressed as mean ± standard error. The number of experimental unit is defined in the methods section and the value of *n* is given in the relevant results section and in figure legends.

## 3. Results

### 3.1. On a Population Level Acute LG Exposure Decreases Hypothalamic but Increases Cortical Astrocyte Intracellular Calcium Levels

**Figure 1:**
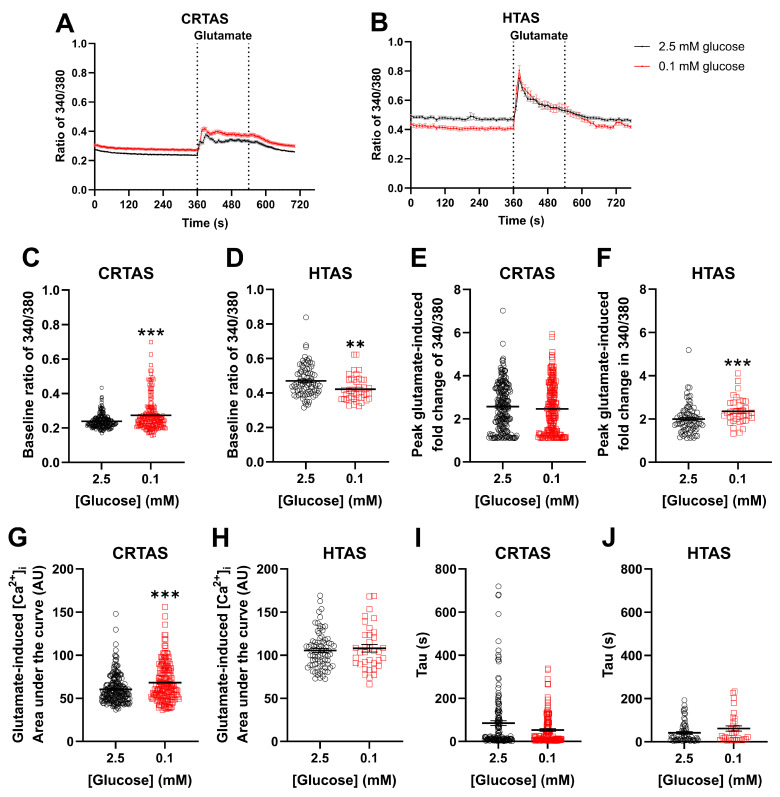
Prior low glucose increases and decreases average basal calcium in cortical and hypothalamic astrocytes respectively. Primary cortical (CRTAS) and hypothalamic (HTAS) astrocytes were loaded with Fura-2 calcium sensitive dye for 1 h in either normal (2.5 mmol/L; CRTAS n = 211 across 13 coverslips; HTAS n = 81 across 8 coverslips, open black circles) or low (0.1 mmol/L; CRTAS n = 186 across 12 coverslips; HTAS n = 42 across 6 coverslips, open red squares) glucose containing normal saline before imaging begun. Intracellular calcium concentration was determined as the ratio of fluorescence emission at 505 nm when stimulated at 340 nm and 380 nm. After 6 min 100 µmol/L glutamate was added for 3 min before washout and termination of imaging. Intracellular calcium levels of CRTAS (**A**) and HTAS (**B**). Baseline ratio of 340/380 in CRTAS (**C**) and HTAS (**D**). Peak glutamate-induced fold change in ratio of 340/380 of CRTAS (**E**) and HTAS (**F**). Glutamate-induced area under the curve in CRTAS (**G**) and HTAS (**H**). Tau, the decay of the glutamate signal, in CRTAS (**I**) and HTAS (**J**). Mann–Whitney tests. Kruskall-Wallis tests with post hoc Dunn’s tests. Two-Way ANOVA with post hoc Dunnett’s multiple comparisons test. ** *p* < 0.01; *** *p* < 0.001.

Astrocytes exhibiting glucose-inhibited behaviour have been demonstrated in glucose-sensing brain regions previously [6,7,8]. We extended these findings by examining the response to acute LG exposure in cultured astrocytes isolated from the hypothalamus and the cortex of neonatal rats. This allowed for comparison between classically glucose-sensing brain nuclei (hypothalamus; HTAS) and a non-glucose sensing region (cortex; CRTAS). It was hypothesised that LG exposure would increase the intracellular Ca^2+^ of HTAS and either not change or decrease calcium in CRTAS. Secondly, given the energetic demands on astrocytes of glutamate uptake and recycling, it was hypothesised that glutamatergic signalling would be impaired during LG exposure in both cell types. To test this, CRTAS and HTAS (Figure 1A,B) were exposed to either normal (2.5 mmol/L; CRTAS n = 211 cells across 13 coverslips; HTAS n = 81 cells across 8 coverslips) or low (0.1 mmol/L; CRTAS n = 186 cells across 12 coverslips; HTAS n = 42 cells across 6 coverslips) glucose for 1 h and Ca^2+^ signals measured. Across the population, basal Ca^2+^ levels were increased in CRTAS (Figure 1C) and decreased in HTAS (Figure 1D) following 1 h of acute low glucose exposure. Glutamate (100 µmol/L) was then added for 3 min before wash-off. 1 h long exposure to low glucose was selected as preliminary studies demonstrated changes to [Ca^2+^]_i_ took approximately 45 min to develop. This is a pathophysiologically relevant timeframe given that individuals with diabetes frequently experience hypoglycaemia lasting up to several hours.

Acute low glucose exposure increased the area under the curve of the Ca^2+^ response to glutamate in CRTAS (Figure 1G) and peak amplitude of glutamate response in HTAS (Figure 1F), otherwise glutamate-induced signalling was minimally impacted. Tau was unchanged in CRTAS or HTAS (Figure 1I,J).

### 3.2. Sub-Populations of Glutamate-Responsive Cortical and Hypothalamic Astrocytes Have a Glucose-Inhibited- or a Glucose-Excited-like Phenotype

**Figure 2:**
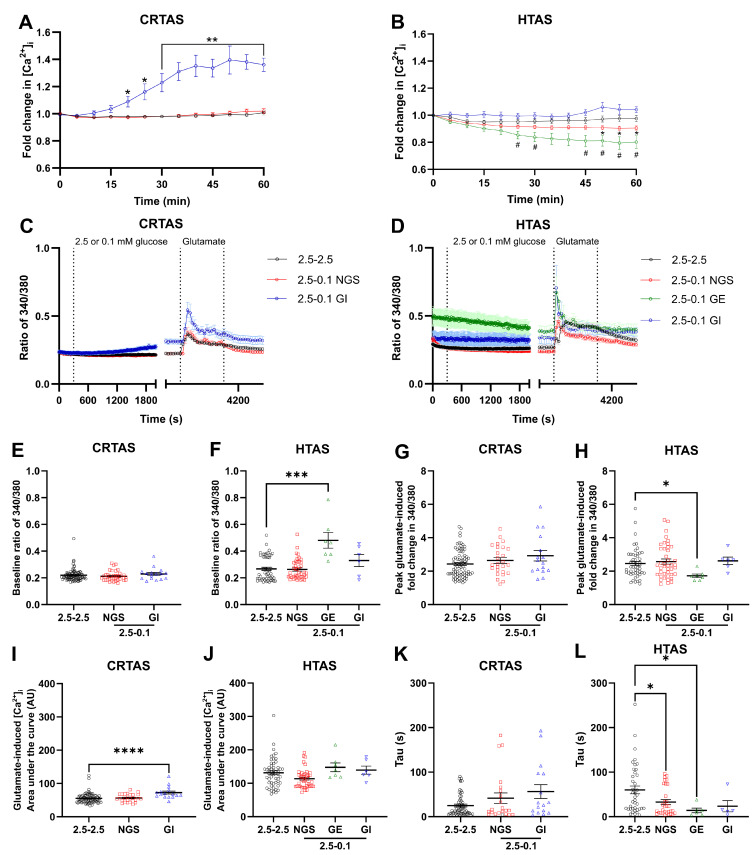
One hour of low glucose changes calcium levels of glucose-sensitive cortical and hypothalamic astrocytes. Primary cortical (CRTAS) and hypothalamic (HTAS) astrocytes were loaded with Fura-2 calcium sensitive dye for 1 h in 2.5 mmol/L glucose-containing normal saline before imaging. After 6 min of imaging in 2.5 mmol/L glucose the cells were either maintained in 2.5 mmol/L glucose (CRTAS n = 83 cells across 7 coverslips; HTAS n = 51 cells across 7 coverslips) or decreased to 0.1 mmol/L glucose (CRTAS n = 44 cells across 6 coverslips; HTAS n = 55 cells across 10 coverslips) for 1 h. Cells were then treated with glutamate (100 µmol/L) for 3 min before washout and termination of imaging. Cells that had a delta in signal from the previous 5 min more than the average of the controls +/−2.5 standard deviations were determined to be glucose-inhibited-like (CRTAS n = 16; HTAS n = 6) or glucose-excited-like (CRTAS n = 2; HTAS n = 7), respectively. As there were less than three glucose-excited-like CRTAS, they were excluded from analysis. The ratio of 340/380 indicates [Ca^2+^]_i_ in CRTAS (**A**), and HTAS (**B**). The ratio of 340/380 values were binned using the 30 s of recording either side of each 5-min time point and displayed as fold change for CRTAS (**C**) and HTAS (**D**). Baseline ratio 340/380 in CRTAS (**E**) and HTAS (**F**). Peak fold-change of glutamate-induced increase in ratio of 340/380 of CRTAS (**G**) and HTAS (**H**). The area under the curve of glutamate-induced increases in [Ca^2+^]_i_ in CRTAS (**I**) and HTAS (**J**). The tau value for the decay in calcium signal induced by glutamate stimulation in CRTAS (**K**) and HTAS (**L**). Error bars represent standard error of the mean. Kruskall-Wallis tests with post hoc Dunn’s tests. Two-Way ANOVA with post hoc Dunnett’s multiple comparisons test. * *p* < 0.05; ** *p* < 0.01; *** *p* < 0.001; **** *p* < 0.0001 from control. # *p* < 0.05 of GE cells from control. Circles, squares, green triangles, and blue triangles denote control, NGS, GE- and GI-like cells, respectively.

To examine in more detail the dynamics of Ca^2+^ responses of CRTAS and HTAS within the population, cells were incubated in 2.5 mmol/L glucose with Fura2 AM for one hour prior to imaging, after baseline recording, glucose levels were lowered to 0.1 mmol/L or maintained in 2.5 mmol/L for one hour. To determine glucose-sensing phenotype, the data were normalised to baseline and data condensed to 1 minute bins every 5 minutes (Figure 2A,B). For both CRTAS and HTAS 55 glutamate-responsive cells were acutely exposed to LG and quantified. In CRTAS 37 (67%) were non-glucose-sensing, 2 were GE-like (4%), and 16 were GI-like (29%) cells. Compared to 42 (76%) non-glucose-sensing, 7 GE-like (13%), and 6 (11%) GI-like cells in HTAS. Therefore, proportionally, the glucose-sensing cells in the CRTAS were more GI-like whereas in the HTAS, the proportion of GE and GI-like cells was similar. The CRTAS GI-like cells had significantly elevated [Ca^2+^]_i_ following 1 h of LG exposure, compared to cells maintained in 2.5 mmol/L glucose (Figure 2A). HTAS GE-like cells significantly decreased [Ca^2+^]_i_ after 1 h of low glucose compared to 2.5 mmol/L glucose treated cells (Figure 2B). While the cells identified as GI-like had increased [Ca^2+^]_i_ after 1 h of low glucose on an individual basis, the mean increase in [Ca^2+^]_i_ was not significantly different from the normal glucose-treated group due to variation within the GI-like cells (Figure 2B). After 50 minutes of LG exposure, non-glucose-sensing HTAS also significantly decreased [Ca^2+^]_i_ compared to normal glucose-treated cells (Figure 2B). This may be due to an inability to separate glucose-sensing cells from the non-glucose sensing group which then affect the mean [Ca^2+^]_i_ level of the group. Alternatively, it may be that even non-glucose-sensing cells decrease [Ca^2+^]_i_ given sufficient time in low glucose due to an inability to sustain the action of Ca^2+^ pumps which consume ATP. There were no significant differences between basal [Ca^2+^]_i_ in CRTAS (Figure 2E). Similarly, in HTAS the non-glucose-sensing and GI-like cells did not have significantly different basal calcium levels from normal glucose-treated cells, whereas the GE-like cells did have elevated basal calcium (Figure 2F).

### 3.3. CRTAS and HTAS Glutamate Responsiveness after One Hour of Normal or Low Glucose

After one hour of normal or LG, glutamate was added for three minutes before wash-off. (Figure 2C,D). Glutamate-induced Ca^2+^ signal amplitude was less in HTAS GE-like cells compared to normal glucose-treated cells (Figure 2H). Interestingly, even though the [Ca^2+^]_i_ of HTAS GE-cells had significantly decreased by the start of the glutamate treatment, it was still relatively higher than other groups, therefore the decreased signal amplitude may be explained by a relatively higher starting [Ca^2+^]_i_ reaching the same peak glutamate-induced response. In CRTAS GI-like cells, the area under the curve (AUC) of glutamate-induced [Ca^2+^]_i_ rises was significantly elevated compared to normal glucose-treated cells (Figure 2I), whereas no significant differences were found in the HTAS cells (Figure 2J). The elevated AUC of glutamate-response in the CRTAS GI-cells may be due to an increase in [Ca^2+^]_i_ prior to the start of the glutamate-treatment period. The time constant (tau) for glutamate signal decay was significantly decreased in HTAS NGS and GE-like cells but remained unchanged in GI-like or CRTAS cells (Figure 2K,L).

### 3.4. Reversibility of LG-Induced Changes in [Ca^2+^]_i_

**Figure 3:**
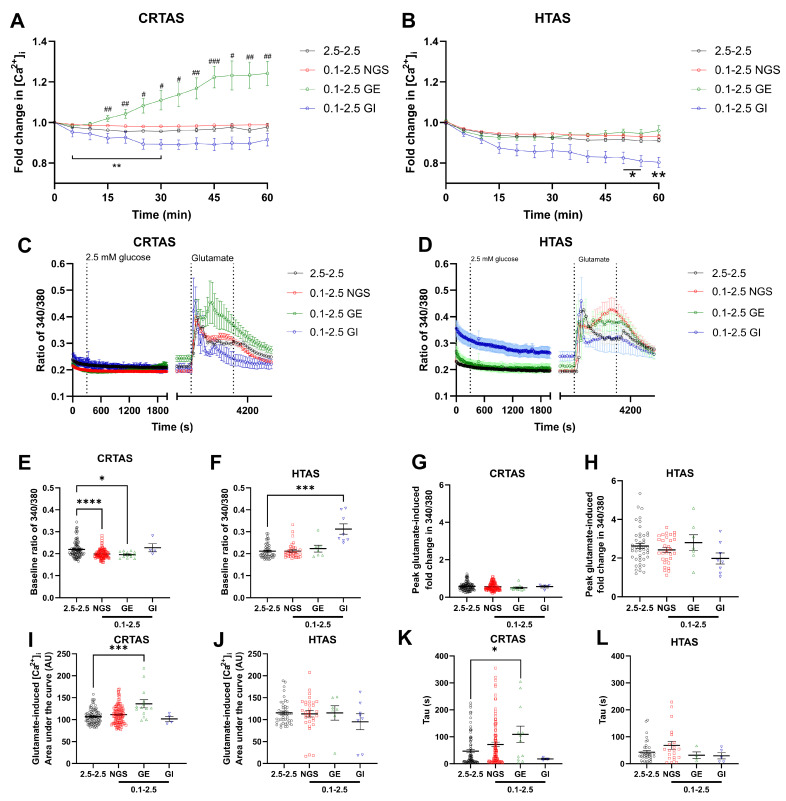
Reversal of low glucose-induced calcium response by return to normal glucose. Primary cortical (CRTAS) and hypothalamic (HTAS) astrocytes were loaded with Fura-2 calcium sensitive dye for 1 h in either 2.5 mmol/L glucose (CRTAS n = 98 cells across 6 coverslips; HTAS n = 48 across 5 coverslips) or 0.1 mmol/L glucose (CRTAS n = 137 cells across 7 coverslips; HTAS n = 36 across 6 coverslips) before calcium imaging. After 6 min of baseline measurement cells glucose levels were maintained or increased to 2.5 mmol/L for 1 h. Cells were then treated with glutamate (100 µmol/L) for 3 min before washout and termination of imaging. Cells that had a delta in signal from the previous 5 min more than the average of the controls +/−2.5 standard deviations were determined to be glucose-excited-like (CRTAS n = 14; HTAS n = 7) or glucose-inhibited-like (CRTAS n = 4; HTAS n = 8), respectively. The ratio of 340/380 indicating [Ca^2+^]_i_ in CRTAS (**A**), and HTAS (**B**). The ratio of 340/380 values were binned using the 30 s of recording either side of each 5-min time point for CRTAS (**C**) and HTAS (**D**). Basal [Ca^2+^]_i_ of CRTAS (**E**) and HTAS (**F**). The fold change of glutamate-induced increase in the ratio of 340/380 of CRTAS (**G**) and HTAS (**H**). The area under the curve of glutamate-induced increases in [Ca^2+^]_i_ in CRTAS (**I**) and HTAS (**J**). The tau value for the decay in calcium signal induced by glutamate stimulation in CRTAS (**K**) and HTAS (**L**). * *p* < 0.05; ** *p* < 0.01; *** *p* < 0.001 **** *p* < 0.0001 denote significant difference between NGS-cells and control or groups designated. # *p* < 0.05, ## *p* < 0.01 and ### *p* < 0.001 denote significant difference between GE-cells and control. Error bars represent standard error of the mean. Kruskall-Wallis tests with post hoc Dunn’s tests. Two-Way ANOVA with post hoc Dunnett’s multiple comparisons test. Circles, squares, green triangles and blue triangles denote control, NGS, GE- and GI-like cells, respectively.

To determine whether acute low glucose-induced changes seen in [Ca^2+^]_i_ were reversible, CRTAS (Figure 3A) and HTAS (Figure 3B) were exposed to 2.5 or 0.1 mmol/L glucose for 1 h during the calcium indicator loading step. Cells were subsequently imaged and perfused for 1 h in 2.5 mmol/L glucose during [Ca^2+^]_i_ measurement. In this experiment, glucose-sensing phenotype was determined by the responses of cells to the increase in glucose concentration from 0.1 to 2.5 mmol/L. In CRTAS, 119 (88%) were NGS, 14 (10%) were GE-like, and 4 (3%) were GI-like cells. In HTAS, 35 (70%) were NGS, 7 (14%) were GE-like, and 8 (16%) were GI-like cells. The CRTAS and HTAS NGS-cells did not significantly alter their [Ca^2+^]_i_ compared to normal glucose-treated cells, whereas GI-like cells significantly decreased [Ca^2+^]_i_ when switched from 0.1 to 2.5 mmol/L glucose (Figure 3A,B). Conversely, the CRTAS GE-like cells increased [Ca^2+^]_i_ in the same conditions, whereas the HTAS GE-like cells were not significantly increased from normal glucose-treated cells. The [Ca^2+^]_i_ at the start of recording, in 0.1 mmol/L glucose, of CRTAS NGS and GE-like cells was significantly lower than normal glucose-treated cells which were in 2.5 mmol/L glucose (Figure 3E), whereas the [Ca^2+^]_i_ of HTAS GI cells was significantly elevated (Figure 3F).

### 3.5. Glutamate Responses of CRTAS and HTAS in Normal Glucose after Low Glucose Exposure

After one hour of normal or low glucose, glutamate was added to the cells for three minutes before wash-off (Figure 3C,D). The peak glutamate-induced change in [Ca^2+^]_i_ was not significantly different between groups in either CRTAS or HTAS (Figure 3G,H). However, the area under the curve of the glutamate-induced change in [Ca^2+^]_i_ was increased in CRTAS GE-like cells compared to normal glucose-treated cells (Figure 3I), whereas the HTAS were unchanged (Figure 3J). The tau of the glutamate-induced calcium signal was increased in GE-like cells in CRTAS (Figure 3K), but in HTAS the tau was not significantly different between groups.

### 3.6. Acute LG Exposure Did Not Affect Glutamate-Induced Changes in Metabolism

**Figure 4:**
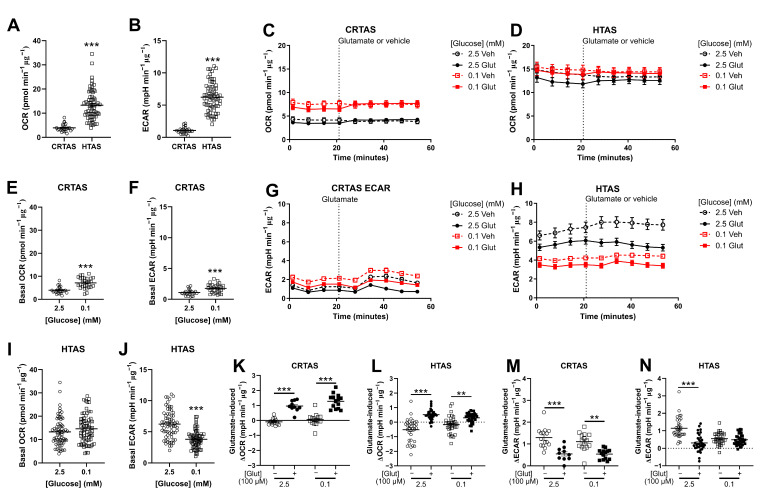
Low glucose does not affect glutamate-induced metabolic changes in primary rat astrocytes. Oxygen consumption rate (OCR) and extracellular acidification rate (ECAR) were measured from CRTAS and HTAS in 2.5 mmol/L glucose before any treatments. HTAS have a significantly higher OCR (**A**) and ECAR (**B**) than CRTAS. Astrocytes were simultaneously treated to 1 h of 0.1 mmol/L glucose and measurements of OCR and ECAR taken before they were treated with vehicle or glutamate (100 µmol/L) (**C**,**D**,**G**,**H**). Basal OCR of CRTAS (**E**). Basal ECAR of CRTAS (**F**). Basal OCR of HTAS (**I**). Basal ECAR of HTAS (**J**). Peak ∆OCR after vehicle or glutamate treatment of CRTAS (**K**) and HTAS (**L**). Peak ∆ECAR after vehicle or glutamate treatment of CRTAS (**M**) and HTAS (**N**). Error bars represent standard error of the mean. Two-tailed unpaired t test. One-way ANOVA with post hoc Tukey’s multiple comparison tests. ** *p* < 0.01; *** *p* < 0.001. CRTAS 2.5 mmol/L glucose with vehicle (n = 15); CRTAS 2.5 mmol/L glucose with glutamate (n = 10); CRTAS 0.1 mmol/L glucose with vehicle (n = 15); CRTAS 0.1 mmol/L glucose with glutamate (n = 14); HTAS 2.5 mmol/L glucose with vehicle (n = 34); HTAS 2.5 mmol/L glucose with glutamate (n = 34); HTAS 0.1 mmol/L glucose with vehicle (n = 35); HTAS 0.1 mmol/L glucose with glutamate (n = 33). Open circles and closed circles represent 2.5 mmol/L glucose with vehicle-treated cells respectively. Open squares and closed squares represent 0.1 mmol/L glucose with glutamate-treated cells respectively.

It was hypothesised that the LG-induced increase in population level [Ca^2+^]_i_ in CRTAS (Figure 1A) would correspond with an increase in mitochondrial and/or glycolytic metabolism to fuel Ca^2+^ handling. Conversely, as LG decreased [Ca^2+^]_i_ in HTAS, a corresponding decrease in cellular metabolism would be expected. Furthermore, as glutamate uptake has a substantial energetic cost [18], and there were differences in glutamate handling by glucose-sensing astrocytes, it was also thought that glutamate-induced cellular metabolic responses would be modulated by LG. To test this, CRTAS and HTAS were incubated in either 2.5 or 0.1 mmol/L glucose for 1 h, then oxygen consumption rates (OCR; indicative of mitochondrial metabolism) and extracellular acidification rates (ECAR; indicative of glycolytic function) were measured. Interestingly, basal OCR and ECAR were substantially and significantly higher in HTAS than CRTAS (Figure 4A,B). Both OCR (Figure 4E) and ECAR (Figure 4F) were increased in CRTAS exposed to 0.1 mmol/L glucose compared to 2.5 mM glucose. In contrast, however, HTAS OCR remained unchanged between normal and LG (Figure 4I), and ECAR was significantly lower in 0.1 mmol/L glucose-treated cells (Figure 4J). To test whether the effects of glutamate on metabolism were altered by LG exposure, glutamate was injected and OCR (Figure 4C,D) and ECAR (Figure 4G,H) were measured for a further 30 min. Compared to vehicle-treated cells, glutamate increased OCR in 2.5 and 0.1 mmol/L glucose and the response was comparable in both CRTAS (Figure 4K) and HTAS (Figure 4L). In both CRTAS (Figure 4M) and HTAS (Figure 4N), glutamate decreased ECAR compared to vehicle in 2.5 mmol/L glucose. In 0.1 mmol/L glucose, the ECAR significantly decreased in CRTAS, but not in HTAS, as these cells already displayed reduced ECAR.

### 3.7. Glutamate Prevention of RLG-Induced Elevated Basal Mitochondrial Respiration in Hypothalamic but Not Cortical Astrocytes

**Figure 5:**
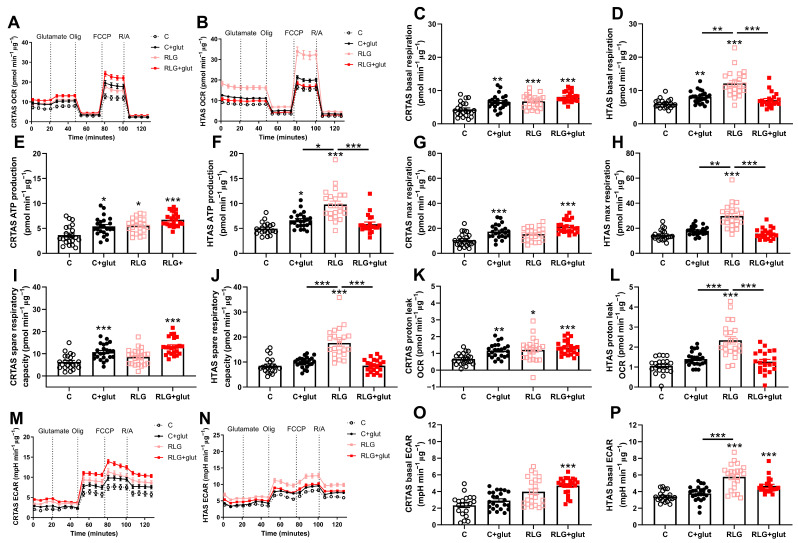
Recurrent low glucose-induced metabolic adaptations attenuated with co-treatment of glutamate in hypothalamic but not cortical astrocytes. Concurrent glutamate treatment exacerbated the effects of RLG in CRTAS on metabolism but ameliorated the effects in HTAS. After control, control plus glutamate (C + glut), RLG or RLG plus glutamate (RLG + glut) treatment, cells underwent a mitochondrial stress test (CRTAS n = 49, 52, 46, 57, respectively, across three separate assays; HTAS n = 21, 24, 22, 21, respectively, across two separate assays). OCR of CRTAS (**A**) and HTAS (**B**). Basal mitochondrial respiration of CRTAS (**C**) and HTAS (**D**) calculated as non-mitochondrial OCR subtracted from basal OCR. ATP-production associated OCR of CRTAS (**E**) and HTAS (**F**), calculated as the difference between basal OCR and ATP-synthase inhibited OCR. Maximal respiratory OCR, induced by the mitochondrial membrane uncoupler FCCP, of CRTAS (**G**) and HTAS (**H**). Spare respiratory capacity of CRTAS (**I**) and HTAS (**J**), calculated as the difference between maximal respiration and basal respiration. Proton leak of CRTAS (**K**) and HTAS (**L**), calculated as the difference between ATP-associated OCR and non-mitochondrial respiration. ECAR of CRTAS (**M**) and HTAS (**N**) during the mitochondrial stress test. Basal ECAR of CRTAS (**O**) and HTAS (**P**). Error bars represent standard error of the mean. * *p* < 0.01; ** *p* < 0.01; *** *p* < 0.001. Normally distributed data were analysed using one-way ANOVAs with post hoc Tukey’s multiple comparisons test. Abnormally distributed data were analysed using Kruskal–Wallis test with post hoc Dunn’s multiple comparisons test. Open circles, closed circles, pink squares and red squares denote control, C + glut, RLG, and RLG + glut, respectively.

RLG induces a reversible increase in mitochondrial respiration in adult human primary astrocytes [13]. However, glutamate recycling by astrocytes has a significant energetic cost and we hypothesised that glutamate would exacerbate the response of astrocytes to RLG by providing additional metabolic burden. Therefore, the effects of RLG with and without concurrent glutamate on cellular metabolism were assessed using mitochondrial stress tests. Glutamate was only added for 3 h during the 0.1 or 2.5 mmol/L glucose incubation on each day. The assay was completed approximately 19 h following the exposure to RLG. Following RLG or control exposure and in the continued presence of 2.5 mM glucose, CRTAS cells exposed to RLG had significantly elevated basal mitochondrial respiration which was not modified by glutamate co-exposure (Figure 5A,C). In HTAS, RLG increased mitochondrial respiration and this was rescued by glutamate co-exposure during RLG (Figure 5B,D). In both cell types, glutamate injection during the mitochondrial stress test had no effect on cellular metabolism which is likely due to the presence of glutamine in the media (Figure 5). This is in contrast to the acute glutamate-induced changes observed, in the absence of glutamine in the media (Figure 4).

In CRTAS, glutamate exposure with and without RLG elevated ATP production-associated OCR, compared to control (Figure 5E). In HTAS (Figure 5F), ATP production-associated OCR was significantly higher following RLG than any other groups. Interestingly, RLG + glut was not significantly different from control indicating prevention of the RLG-induced changes to ATP production (Figure 5F). C + glut and RLG + glut in CRTAS were significantly elevated from controls whereas RLG was not (Figure 5G). In HTAS, the RLG-treated group had significantly elevated maximal mitochondrial respiration compared to the other groups, while neither group pre-treated with glutamate were significantly different from the control (Figure 5H). In CRTAS, C + glut and RLG + glut treated cells had elevated spare respiratory capacity compared to control (Figure 5I). In HTAS, however, RLG alone significantly elevated spare respiratory capacity compared to control and concomitant glutamate exposure during RLG prevented the development of this adaptation (Figure 5J). In CRTAS, C + glut, RLG and RLG + glut groups had significantly increased proton leak from control (Figure 5K), compared to HTAS (Figure 5L) where RLG alone was significantly elevated compared to the other groups. None of the changes to mitochondrial function were mediated by changes to mitochondrial content or the filamentous network structure (ESM Figure S3).

In HTAS the RLG group was also increased basal extracellular acidification rate (ECAR) compared to the control group (Figure 5N,P), with a trend (*p* = 0.073) towards increased ECAR in the CRTAS RLG group (Figure 5M,O). In both CRTAS and HTAS, the RLG + glut groups were significantly elevated from the control suggesting persistently higher rates of basal glycolysis/glucose utilisation following RLG + glut.

### 3.8. RLG Exposure Increases Capacity to Oxidise Glutamine in Hypothalamic Astrocytes

**Figure 6:**
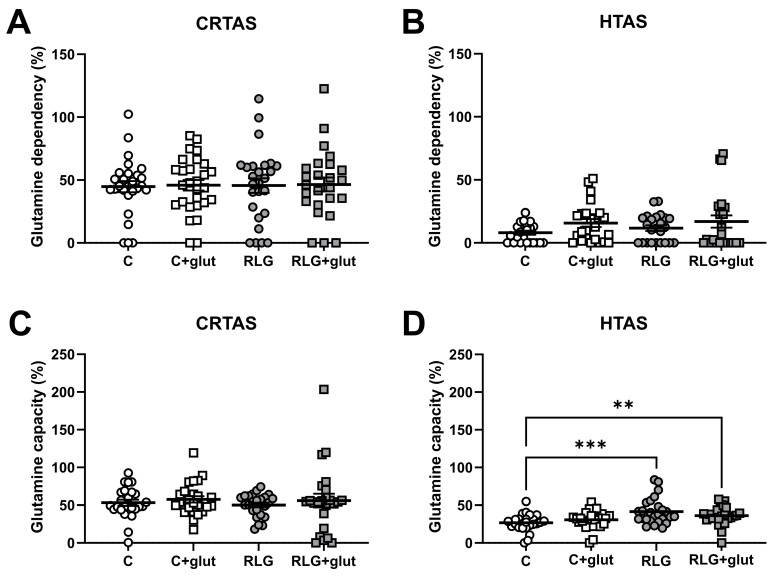
RLG and RLG plus glutamate altered mitochondrial fuel flexibility to increase the capacity to metabolise glutamine. Using pyruvate, fatty acid, and glutamine fuel pathway inhibitors, UK5099, etomoxir and BPTES (bis-2-(5-phenylacetamido-1,3,4-thiadiazol-2yl)ethyl sulfide), respectively, allowed for the measurement of the percentage of which CRTAS and HTAS were dependent on or had the capacity to metabolise glutamine. Dependency: contribution of that pathway to basal OCR. Capacity: maximal ability to consume oxygen through the pathway. CRTAS ((**A**); n = 25–28) and HTAS ((**B**); n = 24–25) dependency on glutamine fuel pathway. CRTAS ((**C**); n = 24–25) and HTAS ((**D**); n = 25–26) capacity to metabolism glutamine fuel pathway. Error bars represent standard error of the mean. ** *p* < 0.01; *** *p* < 0.001. Normally distributed data were analysed using one-way ANOVAs with post hoc Tukey’s multiple comparisons test. Abnormally distributed data were analysed using Kruskal–Wallis test with post hoc Dunn’s multiple comparisons test.3.9. Extracellular glutamate clearance is increased in HTAS following RLG and sustains ATP levels after prolonged low glucose exposure.

Nutrient usage by astrocytes is altered following RLG [13] and people with impaired hypoglycaemia awareness have increased monocarboxylic acid uptake in the brain [31,32]. Therefore, to investigate whether the changes to mitochondrial respiration (Figure 5) were driven by alternative fuel usage, the capacity of and dependency on glutamine and fatty acids as fuels was measured in CRTAS and HTAS. HTAS had a higher capacity for glutamine oxidation after RLG in the absence and presence of glutamate. In contrast, there was no change to glutamine dependency nor capacity in CRTAS (Figure 6A,B). While previous studies in adult human primary astrocytes showed changes in fatty acid dependency, this was not seen in rodent CRTAS or HTAS (ESM Figure S4).

**Figure 7:**
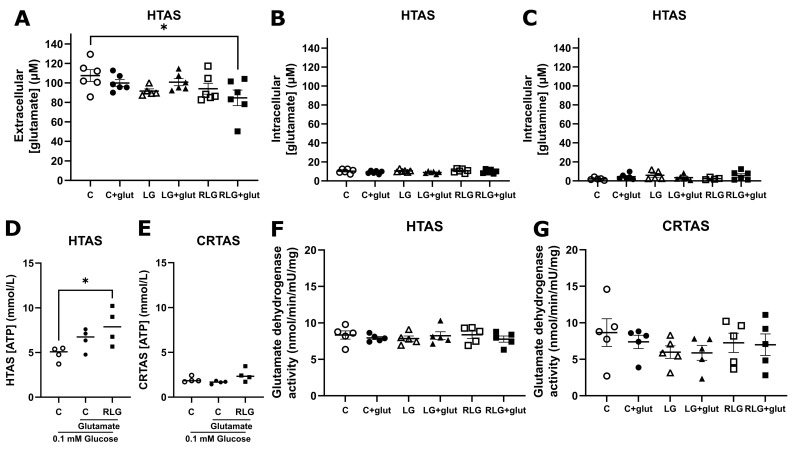
Co-treatment with glutamate in recurrent low glucose increases uptake of glutamate and sustains ATP levels after extended low glucose in hypothalamic astrocytes. After control, acute low glucose (LG) recurrent low glucose (RLG) with and without glutamate HTAS cell lysates and conditioned media were analysed. Extracellular glutamate concentrations ((**A**); n = 5–6). Intracellular glutamate concentrations ((**B**); n = 6). Intracellular glutamine concentrations ((**C**); n = 5–6). ATP levels of HTAS (**D**) and CRTAS (**E**) cellular contents and conditioned media together after exposure to low glucose for 3 h. Activity of intracellular glutamate dehydrogenase activity of HTAS (**F**) and CRTAS (**G**). Error bars represent standard error of the mean. One-way ANOVA with post hoc Tukey’s multiple comparisons test. * *p* < 0.05.

As the capacity to oxidise glutamate-derived glutamine was increased following RLG (Figure 6), it was hypothesised that RLG and RLG + glut treated HTAS may increase their uptake of glutamate. After control or RLG conditions with and without concurrent glutamate treatment, HTAS and CRTAS were exposed to normal or low glucose for 30 min in the presence of glutamate (100 µmol/L). In the conditioned media, RLG + glut treated HTAS had significantly lower extracellular [glutamate] compared to controls (Figure 7A), but intracellular [glutamate] (Figure 7B) and [glutamine] (Figure 7C) were unchanged indicating modest glutamate depletion. There were no changes in CRTAS (ESM Figure S5). In the presence of glutamate RLG treated HTAS cells were able to better defend their intracellular ATP levels following 3 h of low glucose exposure, which was significantly higher than vehicle-treated cells (Figure 7D). There was no difference in CRTAS across the treatment groups (Figure 7E). Taken together these data suggest that following RLG, glutamate-derived glutamine oxidation may contribute to maintenance of ATP levels during subsequent low glucose exposure in hypothalamic but not cortical astrocytes. However, this was not mediated by changes to glutamate dehydrogenase activity (GDH; Figure 7F,G), which was not altered under any experimental condition.

### 3.9. RLG Increases Low Glucose-Induced ATP Release Irrespectively of Glutamate

**Figure 8:**
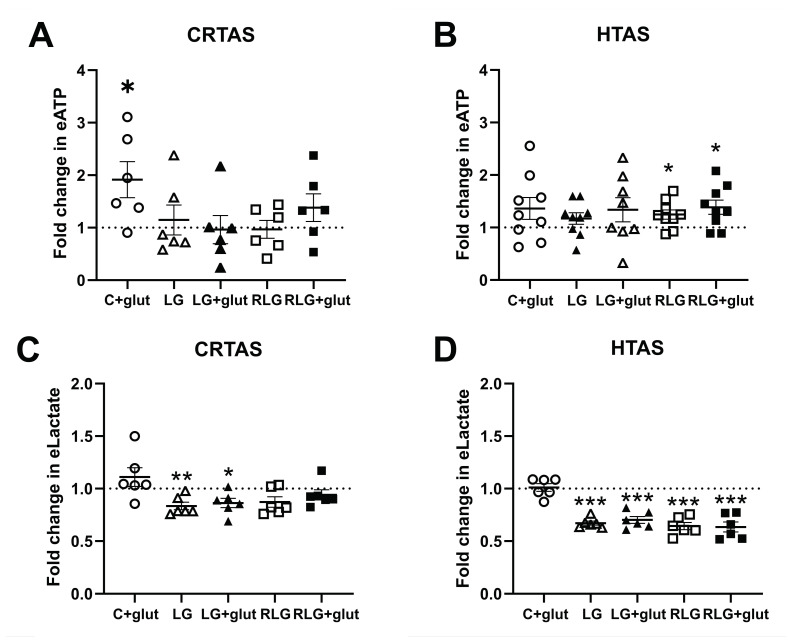
Recurrent low glucose increases ATP release from hypothalamic astrocytes. Recurrent low glucose (RLG) with and without glutamate increased extracellular ATP release from HTAS ((**B**), n = 8–9) but not CRTAS ((**A**), n = 6). Extracellular lactate levels were sustained for 30 min in low glucose by RLG and RLG + glut in CRTAS ((**C**), n = 6) but low glucose conditions decreased HTAS lactate release ((**D**), n = 6). Error bars represent standard error of the mean. * *p* < 0.01; ** *p* < 0.01; *** *p* < 0.001. One-sample *t*-tests, normalised to control.

We next examined whether gliotransmitters were altered in CRTAS and HTAS following RLG. We examined extracellular ATP and lactate, both of which are gliotransmitters known to play a role in hypoglycaemia detection and defective CRR [6,8,33,34]. To test this, extracellular ATP (eATP) and lactate (eLactate) were measured in the conditioned media of CRTAS and HTAS cells exposed to control, C + glut, acute low glucose (LG), LG plus prior glutamate (LG + glut), RLG and RLG + glut treated cells. These cells were also exposed to glutamate for 30 min before the medium was collected. In CRTAS the concentration of eATP was significantly higher in C + glut treated cells than in the control, while the other groups were unchanged (Figure 8A). In the HTAS, however, both RLG and RLG + glut groups, when exposed to glutamate had elevated eATP concentrations compared to control (24.7% and 38.6% respectively; Figure 8B). Therefore, despite low energy availability after RLG and RLG + glut exposure, cells respond to glutamate stimulation by increasing ATP release. In acute low glucose conditions (LG and LG + glut) both CRTAS and HTAS (Figure 8C,D) had significantly decreased extracellular lactate. While CRTAS RLG and RLG + glut treated cells did not significantly decrease lactate levels compared to control, the HTAS RLG and RLG + glut treated cells did.

## 4. Discussion

The blunting of hypoglycaemia-induced CRR is well-known in rats and humans [35,36]. The mechanisms underpinning this phenomenon, however, are incompletely understood. Glutamatergic signalling is involved in the CRR [15] but is diminished in rats after recurrent insulin-induced hypoglycaemia [14], how this occurs requires further investigation. Astrocytes are key contributors to glutamatergic signalling and are responsible for clearing up to 80% of glutamate delivered to the synapse by vesicular release [16,17] as well as recycling glutamine back to neurons to re-use as glutamate. This process is energetically costly, for one glutamate molecule, three sodium ions are co-transported into the cell. The requisite ion-homeostatic mechanisms, including Na^+^/K^+^-ATPase, uses approximately 20% of astrocytic ATP [18]. Hypoglycaemia also disrupts mitochondrial function in the hippocampus and cortex of rats [37] which may disrupt the energy supply for these energetically costly processes. The current study tested the hypothesis that glutamate exposure would alter RLG-induced adaptations by astrocytes. Here, we report that co-supply of glutamate prevents the rise in basal mitochondrial respiration following RLG exposure in hypothalamic but not cortical rat astrocytes. Other studies by our group have shown that human primary astrocytes reversibly adapt to RLG by increasing parameters of mitochondrial respiration and fatty acid metabolism [13]. Another study examining the medio-basal hypothalamus showed changes to genes associated with metabolism after RH [38]. In this study we demonstrated that in HTAS, glutamate exposure during the acute bouts of low glucose, led to defence of intracellular ATP levels compared to non-glutamate-treated cells. Additionally, RLG plus glutamate exposure in HTAS increased the capacity to metabolise glutamate/glutamine and clear glutamate from the extracellular space. While we found no changes to glutamate dehydrogenase activity, which converts glutamate to α-ketoglutarate to enter the TCA cycle, astrocytes in low glucose have been reported to increase glutamate utilisation as a fuel for oxidative phosphorylation [25]. Therefore, while both CRTAS and HTAS adapted to RLG in a similar manner, their adaptation in the presence of glutamate was different. These data support the notion that HTAS increase their use of glutamate as an alternative fuel source during low glucose exposure, thus reducing the necessity for mitochondrial adaptations. These findings are in line with and add to the previous findings of impaired glutamatergic signalling after RH in vivo [14].

The mechanisms by which CRTAS and HTAS have different responses to RLG (and to glutamate co-supply) are not completely clear. It will be interesting in future studies to partition the glutamate receptor mediated responses from the transport/metabolic responses. Our data add to the growing volume of information on astrocytic heterogeneity. For example, hypothalamic but not cortical mouse astrocytes suppress palmitate oxidation when glucose is present [39]. Furthermore, hypothalamic astrocytes are involved in sensing and regulating hormones and nutrients including glucose [4], lipids [40,41], ketones [42,43], insulin [44] and leptin [45,46]. For the first time, the data here demonstrate some notable differences in metabolism between CRTAS and HTAS; the mitochondrial and glycolytic rates, and the intracellular ATP levels are higher in HTAS than CRTAS, whereas the intracellular glutamate and glutamine levels, as well as dependency on and capacity to metabolise glutamine is lower in HTAS than CRTAS. Perhaps because HTAS have a lower intracellular level of glutamate and glutamine, there is capacity for increased metabolism of these amino acids following RLG. By what mechanism these adaptations take place is not known, but it is not due to changes to the activity of glutamate dehydrogenase. While there were no changes in glutamate-induced cellular metabolic changes during acute low glucose, the overall metabolic response of CRTAS and HTAS was different. In low glucose, CRTAS increase both mitochondrial respiration and ECAR (an indicator of glycolysis), whereas HTAS mitochondrial respiration remained the same and ECAR decreased. This may be, at least in part, explained by, on a population level, [Ca^2+^]_i_ increasing in CRTAS when exposed to one hour of low glucose, compared to HTAS in which it decreased. In both cell types, extracellular lactate levels decreased in low glucose, as we only include glucose, glutamine, and pyruvate as standard carbon courses in our experiment. However, this drop (approx. 25–40%) was much less than the 25-fold reduction in extracellular glucose (2.5 vs. 0.1 mM), suggesting a relative increase in lactate production from per mole of glucose available or from low glucose-induced breakdown of glycogen stores [47]. Previous studies have demonstrated increased basal hypothalamic lactate levels following RH in rats [34], which we did not observe. This may be an artefact of the in vitro model, where other carbon sources including other monoamines, amino acids, fatty acids and ketones are available to support lactate production.

Further examination of LG-induced [Ca^2+^]_i_ responses in CRTAS and HTAS indicated that during transitions from normal (2.5 mmol/L) to low (0.1 mmol/L) and from low to normal glucose levels there were a higher percentage of GI-like cells in CRTAS compared to HTAS. GI-like cells increased [Ca^2+^]_i_ in LG at a rate two and a half standard deviations greater than cells exposed to only normal glucose. Likewise, GE-like cells decreased intracellular calcium at a rate two and a half standard deviations greater than cells exposed to only normal glucose. Others have shown astrocytes in ex vivo brain slices of the hypothalamus and hindbrain increase [Ca^2+^]_i_ levels in response to low glucose/glucoprivation, additionally without this response the surrounding neurons fail to respond to low glucose [48]. Our data show retained glucose-responsive changes to [Ca^2+^]_i_ in isolated astrocytes. These changes take longer to appear compared to those seen in brain slices induced by 2-deoxyglucose [6,7,8] which take 5–15 min, which appear more rapidly. The descrepancy may be due to the energy demands from neuronal activity which may accelerate this astrocytic energy depletion. Taken together this supports the case for heterogeneous astrocyte populations that modulate Ca^2+^ signalling in a glucose-dependent fashion.

As mentioned previously, astrocytes at glutamatergic synapses sequester large amounts of glutamate, which presents a significant energetic challenge to the cell due to the maintenance of ion homeostasis [22]. It was hypothesised that glutamate-induced signalling would be altered by the challenge of LG, which in itself represents a metabolic challenge. In both CRTAS and HTAS the acute intracellular Ca^2+^ response to glutamate was largely intact at low glucose levels. However, GE-like cells had either a reduced amplitude of response to glutamate or an increased decay in response signal in low glucose, compared to normal glucose, indicating they were less able to sustain a robust response to glutamate or to terminate the glutamate-induced change as efficiently. Conversely, CRTAS GI-like cells in LG had an increased glutamate-induced area under the curve, compared to normal glucose, indicating a sustained response to glutamate. Together, this indicates that the glucose-sensing phenotype of the cells affected the glutamate responses, albeit only modestly. These responses could be mediated by activation of metabotropic (i.e., mGluR5 [20]) or ionotropic glutamate receptors (i.e., AMPA) which are widely expressed in astrocytes [19]. Nevertheless, increased glutamate-induced Ca^2+^ transients can result in the release of gliotransmitters which in turn modulates local neural cell activity [49,50]. For example, in NTS-containing brain slices astrocytic purinergic signalling is required for a robust glucose-sensing response in neurons [8]. Therefore, glutamatergic signalling resulting in astrocytic Ca^2+^ transients may contribute to purinergic signalling from astrocytes and impact glucose-sensing and the CRR to hypoglycaemia. Furthermore, if after RLG astrocytes are sequestering more glutamate and using it as a fuel with relatively less being recycled for neurotransmission, this could in turn affect low-glucose-induced glutamate and purinergic signalling. However, in the present study and in contrast to expectations, ATP release in response to glutamate was increased from HTAS previously exposed to RLG.

In conclusion, this study shows the presence of sub-populations of GI- and GE-like astrocytes in cultures of HTAS and CRTAS which correspond with altered responsiveness to glutamate in low glucose. Secondly, we identified key differences between CRTAS and HTAS, with HTAS having a higher basal metabolism than CRTAS, but CRTAS have higher intracellular glutamate and glutamine levels. Lastly, this work provides evidence that increased basal mitochondrial metabolism induced by RLG are prevented by the co-supply of glutamate during low glucose in hypothalamic but not cortical astrocytes. Whether this phenomenon is part of the failure of glutamatergic signalling in vivo after RH remains to be determined. Future work should identify whether the adaptations demonstrated here persist in a more replete model such as ex vivo brain slices or in vivo and explore their impact on astrocyte-neuron communication and glucose-sensing. By better understanding the mechanisms involved in adaptations to RLG new therapeutic targets may emerge to prevent blunting of CRR.

## Data Availability

The data presented in this study are available on request from the corresponding author. The data are not publicly available due to privacy or ethical restrictions.

## Supplementary Materials

The following supporting information can be downloaded at: https://www.mdpi.com/article/10.3390/cells11213422/s1, Figure S1: GFAP is expressed in greater than or equal to 90% of isolated cells and cells also express EAAT1 and EAAT2; Figure S2 Model of recurrent low glucose protocol; Figure S3: Mitochondrial network is unaffected by RLG; Figure S4: Fatty acid dependency or capacity is unchanged in rat hypothalamic or cortical primary astrocytes; Figure S5: Recurrent low glucose with and without concurrent glutamate treatment does not alter the amount of extracellular or intracellular glutamate, or intracellular glutamine; Figure S6: Basal intracellular calcium is on average higher in hypothalamic than cortical rat primary astrocytes.
