# Supplementary material for: Glutamate Prevents Altered Mitochondrial Function Following Recurrent Low Glucose in Hypothalamic but Not Cortical Primary Rat Astrocytes
Source: Cells. 2022 Oct 29;11(21):3422. doi: 10.3390/cells11213422 (PMC9654357; doi:10.3390/cells11213422)
Supplement: Supplementary file 1 [file cells-11-03422-s001.zip › cells-1888451-SM.pdf]

Extra Supplementary Methods

Isolation of primary astrocytes

The outer cortex and hypothalamus were isolated from neonatal rats 1-5 days after birth by microdissection, with careful attention to remove the meninges, homogenised with a scalpel and digested in trypsin (0.5% v/v) in HBSS (Ca^{2+}/Mg^{2+}-free). Trypsin was neutralised by the addition of media containing FBS. Tissues were further dissociated with mechanical dissociation using a pipette. Suspensions were filtered using a 70 µm sterile filter and cells were plated onto poly-L-lysine coated T75 flasks with 10 mL of stock medium. The next day, media was changed and subsequently changed every 2-3 days until confluent. At least 24 hours before experimentation cell medium was supplemented with a further 200 µmol/L of dibutyryl-cyclic adenosine monophosphate (d-cAMP). For experiments, cells were seeded at the following densities: standard 96-well cell culture plates contained 20,000 cells per well; 35 mm dishes contained 200,000 cells; T25 culture flasks contained 250,000 cells; 60 mm dishes contained 450,000 cells and both T75 culture flasks and 100 mm dishes contained 750,000 cells.

Analysis of cellular metabolism

The night before experimentation, cells were seeded into XFe96 cell culture plates containing 20,000 cells per well and incubated overnight in stock media containing 200 µmol/L d-cAMP. The cartridge was re-hydrated according to manufacturer's instructions. Cells were kept in a humidified atmospheric incubator at 37 °C for 1 h before extracellular flux analysis by the Seahorse XF^e96 analyser. Low buffered basal media was used (#103575-100, Agilent) and supplemented with 2.5 mmol/L glucose or 0.1 mmol/L glucose, and 2.5 mmol/L pyruvate and adjusted to pH 7.40 at 37 °C. Note, glutamine was excluded from the medium for glutamate treatment of 100 µmol/L. For mitochondrial stress tests and fuel flex tests, glutamine was added at 2 mmol/L. Following equilibration/calibration, measurements were taken every six minutes on a three-minute mix: three-minute measure cycle.

Mitochondrial stress tests were completed as instructed by the manufacturer. Final well concentrations of the mitochondrial inhibitors used (µM): oligomycin 1, FCCP 1, rotenone and antimycin 0.5.

Fuel flex tests were completed as instructed by the manufacturer. Final well concentrations of inhibitors used (µM): BPTES 3, etomoxir 4, UK5099 2.

Single-cell calcium imaging

Cells were loaded with Fura-2 AM (4 µmol/L; Life Technologies) for one hour in HEPES-buffered normal saline containing either 2.5 or 0.1 mmol/L glucose depending on the experimental paradigm. Before imaging began Fura-2 AM-containing normal saline was removed and replaced with fresh saline containing the same glucose concentration. Imaging was performed using a TE 2000-S Eclipse microscope (Nikon), using the 79001-ET Fura2 filter set (Chroma), the lamda DG4 light source (Sutter Instrument Company), and an ORCA-ER digital camera (Hamamatsu). Throughout imaging, cells were continuously perfused in a low volume chamber and ratiometric calcium imaging was performed using pair-wise exposures at 340 and 380nm and analysed using Volocity software. Post hoc analysis was performed using GraphPad Prism and Microsoft Excel.

Categorisation of glucose sensing cells:

1. If a cell had a >= 10% increase in calcium in the window of glutamate addition, then they were determined to be glucose sensing. Non-responders were excluded from further analysis
2. 1 minute of data was averaged at 0, 5, 10,...55 and 60 minutes of glucose treatment. This was from 30 seconds before to 30 seconds after the time point, i.e. 14:30-15:30 for the 15 minute time point.

3. The change in 340/380 ratio for every 5 minute interval was calculated by subtracting the timepoint of interest from the preceding time point, for example, to work out the change in calcium at 60 mins from 55 mins = (ratio of 340/380 at 60 mins) - (ratio of 340/380 at 55mins). This was done to account for any general trend for cells regardless of glucose treatment. For example, signal bleaching occurs during long-duration imaging process so the signal will decrease over time. Or the cells increase in stress throughout the imaging process and their calcium increases.
4. To then identify whether a cell was glucose-sensing or not, its rate of change from the previous timepoint was compared against the average for the 2.5-2.5 mmol/L glucose-treated cells (control). This allowed mitigation of non-glucose mediated changes in calcium signal, such as photobleaching. If the rate of change was ≤ or ≥ (the average of control + (2.5 x Standard deviation of the control cells at that time point) for glucose-excited and glucose-inhibited cells, respectively.
5. The control (2.5-2.5), 0.1-2.5 non glucose sensing cells (NGS), and 0.1-2.5 glucose excited (GE) cells were separated into groups for downstream analysis.
6. Fold-change data were generated using the 0 min as the starting point, following a 5 mins equilibration period to allow for stabilisation of baseline. These data were separated based on the filtering of the raw data.

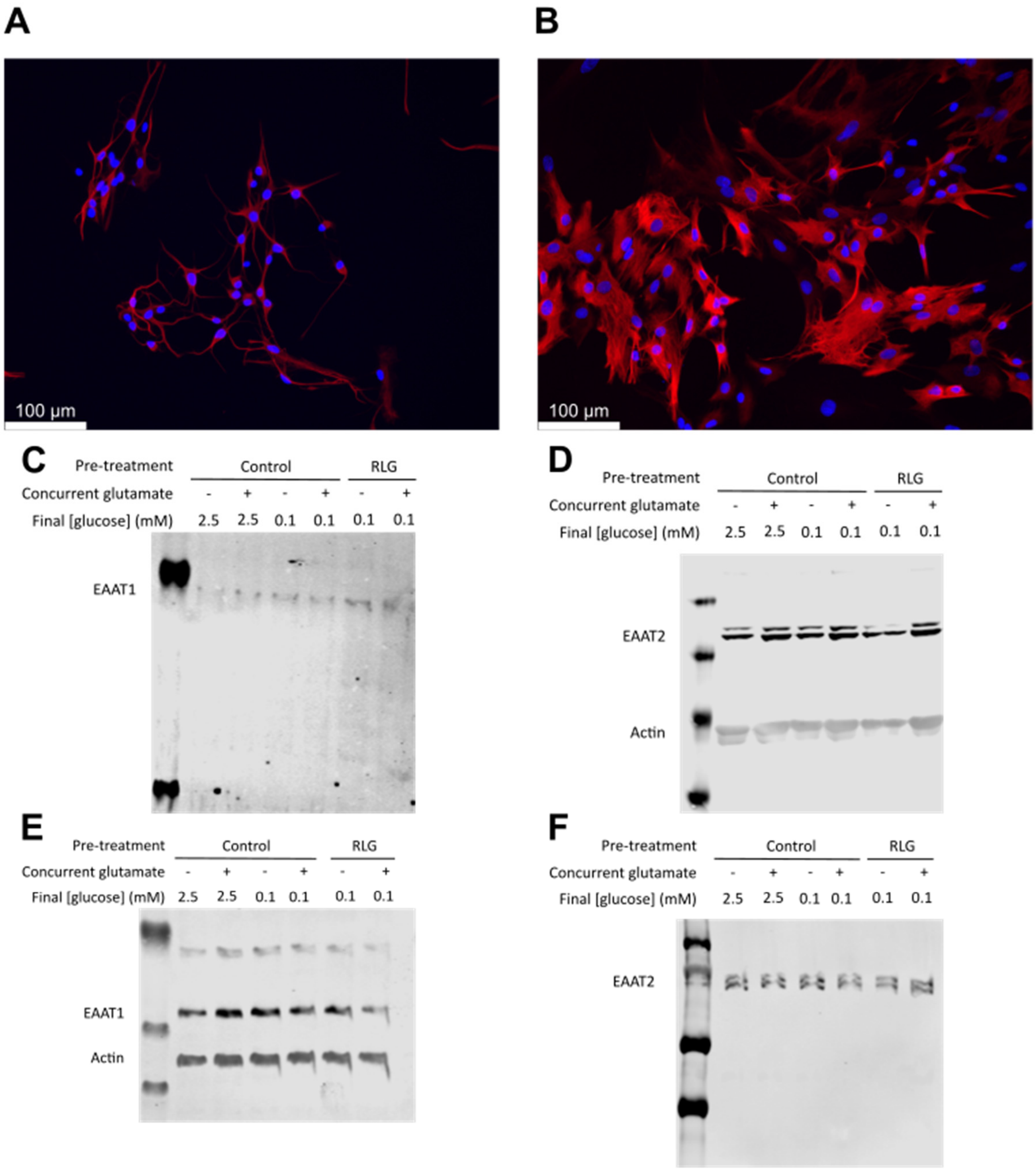

Figure S1. GFAP is expressed in greater than or equal to 90% of isolated cells and cells also express EAAT1 and EAAT2.Both cortical (CRTAS; from 4 separate preparations) and hypothalamic (HTAS; from 3 separate preparations) rat primary astrocytes are greater than or equal to 90% GFAP positive as calculated by manual counting. CRTAS (**A**) and HTAS (**B**) cells stained with a GFAP antibody (red) and DAPI to identify nuclei (blue). Scale bar: 100 µm. CRTAS were exposed to control, acute low glucose, and recurrent low glucose (RLG) with and without 100 µM glutamate treatment. **C, D.** Representative immunoblots for EAAT1 (n=2), EAAT2 in CRTAS (n=4), respectively. **E, F.** Representative immunoblots for EAAT1 (n=4), EAAT2 in HTAS (n=4), respectively.

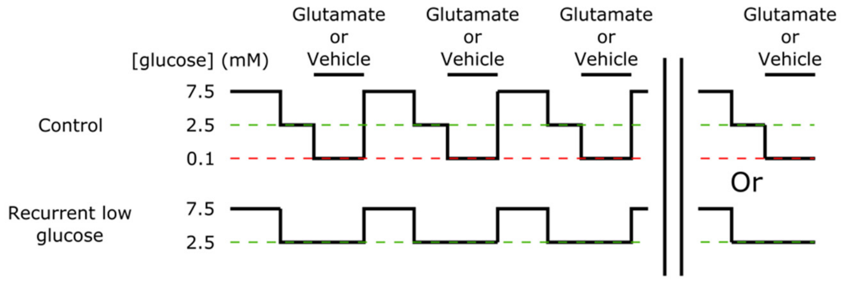

Figure S2. Model of recurrent low glucose protocol. After isolation, cortical and hypothalamic astrocytes were kept in culture for 3-10 days, changing media every 1-3 days. Difference in time in culture was due to differences in seeding density. When cells reached 70-90% confluency, 200 μmol/L dibutyryl cyclic-adenosine monophosphate (d-cAMP) was added to the cells and media changed every 24 hours until cells were seeded into the necessary culture vessel. Cells were cultured in stock media containing 7.5 mM glucose and d-cAMP overnight. Each day for three days cells would be washed and incubated in 2.5 mmol/L glucose containing medium for 2 hours. For the next 3 hours cells would be cultured in 2.5 or 0.1 mmol/L glucose. Cells would then be recovered overnight in stock media. On the third day in some experiments, the cells would be recovered for 2 hours in stock media before being split into an experimental culture vessel for the fourth day. On the fourth day, cells would again be further exposed to either 2.5 or 0.1 mmol/L glucose as described above. This generated the following groups; control, which only had 2.5 mmol/L glucose, acute low glucose, which had 3 days of 2.5 mmol/L and on the last day one exposure to 0.1 mmol/L glucose, and recurrent low glucose, which had 4 exposures to 0.1 mmol/L glucose. For each of the three groups they were also treated to either vehicle (water) or 100 μmol/L glutamate. This was added during the 3 hour incubation each day. Together this generated six groups in total, control, control plus glutamate, acute low glucose, acute low glucose plus glutamate, recurrent low glucose, and recurrent low glucose plus glutamate. Finally, for measurements of oxygen consumption rate and extracellular acidification rate, after the fourth day cells were seeded into Seahorse Bioanalyser cell culture plates for experimenting on the following day.

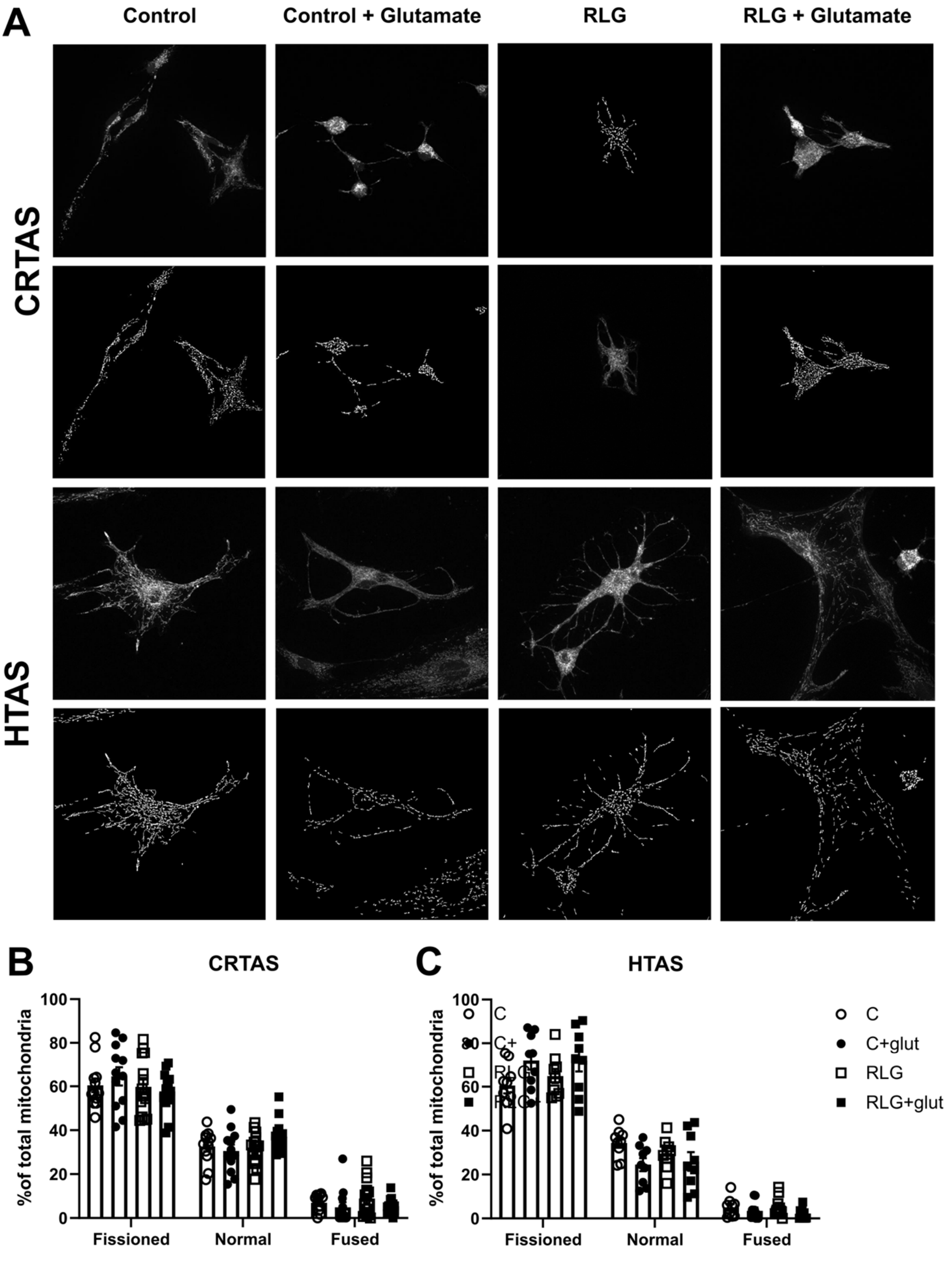

Figure S3. Mitochondrial network is unaffected by RLG. Acute and recurrent low glucose did not alter mitochondrial number or length. **A.** Representative confocal images of cortical (CRTAS) and hypothalamic (HTAS) astrocytes cells exposed to 2.5 or 0.1 mmol/l glucose for 3 hours following control or recurrent low glucose exposure (RLG), with and without glutamate. Raw confocal images are shown in the first and third rows, with extracted signal images shown in the second and fourth rows. **b,c.** Quantification of median object size (in pixels) using a custom MatLab script (**B**, CRTAS 12-15 images across 4-5 experiments; **C**, HTAS 9 images across 3 separate experiments). One-way

ANOVA with post hoc Tukey's multiple comparisons tests. Error bars represent the standard error of the mean.

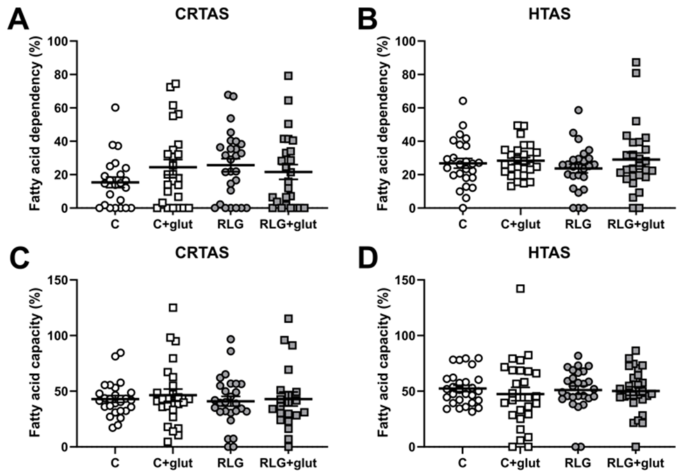

Figure S4. Fatty acid dependency or capacity is unchanged in rat hypothalamic or cortical primary astrocytes. Using pyruvate, fatty acid, and glutamine fuel pathway inhibitors, UK5099, etomoxir and BPTES (bis-2-(5-phenylacetamido-1,3,4-thiadiazol-2yl)ethyl sulfide) respectively allowed for the measurement of the percentage of which CRTAS and HTAS were dependent on or had the capacity to metabolise fatty acids. Dependency: contribution of that pathway to basal OCR. Capacity: maximal ability to consume oxygen through that pathway. CRTAS (**A**; n=23-27) and HTAS (**B**; n=27-28) dependency on fatty acid fuel pathway. CRTAS (**C**; n=21-27) and HTAS (**D**; n=25-27) capacity to metabolism fatty acid fuel pathway. Error bars represent standard error of the mean. Normally distributed data waere analysed using one-way ANOVAs with post hoc Tukey's multiple comparisons test. Abnormally distributed data were analysed using Kruskal-Wallis test with post hoc Dunn's multiple comparisons test.

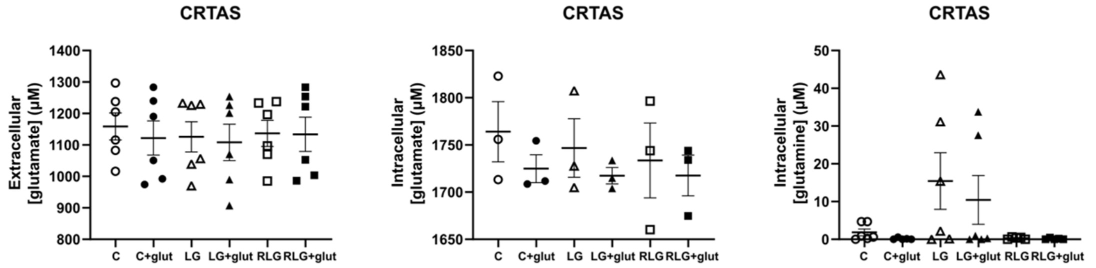

Figure S5. Recurrent low glucose with and without concurrent glutamate treatment does not alter the amount of extracellular or intracellular glutamate, or intracellular glutamine. After control, acute low glucose (LG) recurrent low glucose (RLG) with and without glutamate HTAS cell lysates and conditioned media were analysed. Extracellular glutamate concentrations (**A**; n=6). Intracellular glutamate concentrations (**B**; n=3). Intracellular glutamine concentrations (**C**; n=6). Error bars represent standard error of the mean. One-way ANOVA with post hoc Dunnett tests.

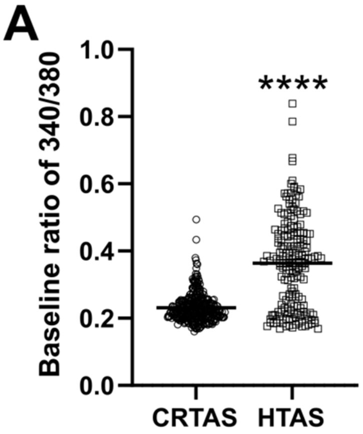

Figure S6. Basal intracellular calcium is on average higher in hypothalamic than cortical rat primary astrocytes. Cortical (CRTAS) and hypothalamic (HTAS) astrocytes were incubated in 2.5 mmol/L glucose containing normal saline for 1 hour whilst loading with Fura2 AM. The baseline reading was taken after five minutes of imaging to establish a stable baseline. Basal intracellular calcium of CRTAS (n= 349 cells) and HTAS (n=187 cells). Error bars represent standard error of the mean. Mann-Whitney test.
